# The evaluation of IMR in crisis resolution home treatment, a mixed methods study protocol

**DOI:** 10.1192/j.eurpsy.2023.305

**Published:** 2023-07-19

**Authors:** S. Kieft, I. Schaap

**Affiliations:** Emergency psychiatry, GGZ inGeest, Amsterdam, Netherlands

## Abstract

**Introduction:**

In the past years, recovery has become a central concept in psychiatric treatment (Sowers *et al*. Acad Psychiatry 2016; 40 461-467). However, in acute mental health care the concept of recovery is lacking attention (Rabenschlag *et al.* Psychiatry Q 2014; 85 225-239; Jaeger *et al.* Nord J Psychiatry 2015; 69 188-195; Luigi *et al*. Can. Med. Educ. J. 2010; 11 62-73). In 2021 a short version of Illness Management and Recovery (IMR) was implemented at the Psychiatric Emergency Service Amsterdam-Amstelland (SPAA). IMR is an evidence based group intervention for patients with severe mental illness, based on cognitive-behavioral, psychoeducational and motivational components. The aim of IMR is to support participants to manage their mental illness (Mueser *et al*. Schizophr Bull 2006; 32 32-43). To our knowledge this the first time that IMR is implemented within an acute mental health care setting Therefore the effects of IMR program on recovery in the acute phase of psychiatric illness are unknown.

**Objectives:**

Insight in effects of IMR in acute mental health care.

**Methods:**

We will carry out a mixed method study. In phase 1 the intervention will be carried out. 25 patients who are admitted to acute mental health care and diagnosed with a severe mental illness (SMI) will take part in the shortened version of IMR. Effects will be measured by the client version of the IMR-scale (Salyers *et al*. Community Ment. Health J. 2007; 43 459-480).Phase 2 includes qualitative interviews with a subsample from phase 1 (using maximum variety in diagnosis and demographic characteristics) to gain insight in the mechanisms and impact of the program.

**Results:**

The proposed study will investigate the effects of an adjusted evidenced-based treatment within a population of people who receive treatment in a Psychiatric Emergency Service. The original intervention is shortened in time and topics, to match the needs of people in the acute phase of psychiatric illness. The question that arises is if an existing treatment can be translated to a different group of patients.

**Conclusions:**

The proposed project has some important limitations that we feel deserve mentioning. It is questionable if a person can profit from a recovery program during a phase of acute crisis. Also, can we expect the same effects if the new program is a shortened version of the original evidence based intervention?

**Disclosure of Interest:**

None Declared

